# Non-specific filtering of beta-distributed data

**DOI:** 10.1186/1471-2105-15-199

**Published:** 2014-06-19

**Authors:** Xinhui Wang, Peter W Laird, Toshinori Hinoue, Susan Groshen, Kimberly D Siegmund

**Affiliations:** 1 Department of Preventive Medicine, USC Keck School of Medicine, University of Southern California, 2001 N Soto Street, Suite 202W, Los Angeles 90089-9239 California, USA; 2 Epigenome Center, USC Keck School of Medicine, University of Southern California, Los Angeles, CA, USA

## Abstract

**Background:**

Non-specific feature selection is a dimension reduction procedure performed prior to cluster analysis of high dimensional molecular data. Not all measured features are expected to show biological variation, so only the most varying are selected for analysis. In DNA methylation studies, DNA methylation is measured as a proportion, bounded between 0 and 1, with variance a function of the mean. Filtering on standard deviation biases the selection of probes to those with mean values near 0.5. We explore the effect this has on clustering, and develop alternate filter methods that utilize a variance stabilizing transformation for Beta distributed data and do not share this bias.

**Results:**

We compared results for 11 different non-specific filters on eight Infinium HumanMethylation data sets, selected to span a variety of biological conditions. We found that for data sets having a small fraction of samples showing abnormal methylation of a subset of normally unmethylated CpGs, a characteristic of the CpG island methylator phenotype in cancer, a novel filter statistic that utilized a variance-stabilizing transformation for Beta distributed data outperformed the common filter of using standard deviation of the DNA methylation proportion, or its log-transformed M-value, in its ability to detect the cancer subtype in a cluster analysis. However, the standard deviation filter always performed among the best for distinguishing subgroups of normal tissue. The novel filter and standard deviation filter tended to favour features in different genome contexts; for the same data set, the novel filter always selected more features from CpG island promoters and the standard deviation filter always selected more features from non-CpG island intergenic regions. Interestingly, despite selecting largely non-overlapping sets of features, the two filters did find sample subsets that overlapped for some real data sets.

**Conclusions:**

We found two different filter statistics that tended to prioritize features with different characteristics, each performed well for identifying clusters of cancer and non-cancer tissue, and identifying a cancer CpG island hypermethylation phenotype. Since cluster analysis is for discovery, we would suggest trying both filters on any new data sets, evaluating the overlap of features selected and clusters discovered.

## Background

Non-specific filtering of variables, the selection of a subset of variables based on a characteristic unrelated to the outcome of interest, is often applied for dimension reduction in high-dimensional data sets. The approach can be used for both supervised and unsupervised analysis. For differential expression, non-specific filtering of features prior to hypothesis testing can increase power to detect differentially expressed genes [1]. For cluster analysis, it can improve sensitivity of finding disease clusters [2]. The most common filter methods use a measure of variance to rank variables, hoping to enrich for features that vary due to biological signal [3-5]. In these settings, the variance of the (possibly log-transformed) data is usually independent of the mean. However, when studying DNA methylation measured as a proportion, this may not always be the case, and alternate filter statistics may be preferred.

DNA methylation is an epigenetic mark that varies between different cell types, correlating with DNA packaging within the cell and facilitating cell-type specific function. Today, Illumina’s DNA methylation BeadArrays allow for high-throughput analysis, with their most recent platform measuring hundreds of thousands of targeted loci for large numbers of samples. On Illumina’s platform, DNA methylation is measured by the percentage of total fluorescence due to methylation. This value is bounded between 0 and 1, and can be modelled using a Beta distribution. To perform cluster analysis on such data, Houseman et al. (2008) [5] developed a recursive partitioning mixture model (RPMM) for Beta-distributed data. They applied a non-specific filter prior to cluster analysis, using the standard deviation of the Beta values. However, for Beta-distributed data, the variance is a function of the mean, and a standard deviation filter will bias the selection of most variable features towards those having a mean near the middle of the 0 to 1 scale. This bias could favour selecting features that show cell-type specific methylation and be desirable for clustering subgroups of normal tissue, as CpG methylation at cell-type specific marks can be sensitive to shifts in the distributions of cells driving the associations of CpG methylation with sample characteristic (e.g. disease state, or age) [6-8]. However, at the same time, a bias that favours selecting features with mean DNA methylation levels near 0.5 can be problematic in studies to discover cancer subtypes where aberrant DNA methylation may only be observed in a small fraction of tumors [9,10]. When the subset of tumors with aberrant profiles is rare, the average DNA methylation level across a set of tumors for sites that are normally unmethylated is closer to the normal state of 0 than 0.5. At the same time, the average level for sites that are normally methylated is closer to 1. When filtering variables based on standard deviation, clusters having only a few samples may not separate distinctly from the rest. To decrease the association between the mean and variance of methylation proportions measured on the Illumina platform, Du et al. (2010) [11] propose using a logit transformation (on the log2 scale). We explore alternate transformations that take the Beta-distribution explicitly into account. In particular, we consider methods making use of the cumulative distribution function, the variance stabilizing transformation for a Beta distribution [12].

We compare different filtering methods in a collection of real data sets generated on either Illumina’s HumanMethylation27 or HumanMethylation450 platform. The variety of examples considered will allow us to evaluate filter methods across different data distributions and structures. We find that the properties of the data set, specifically the fraction of samples in a subtype, or the variation of features within groups, can lead to very different behaviour of some filtering methods.

## Methods

### Illumina HumanMethylation BeadArrays

Illumina’s BeadArray technology analyzes more than 27,000 targeted CpGs on the HumanMethylation27 (HM27) platform, and over 480,000 on the HumanMethylation450 (HM450) [13]. Whereas the HM27 array primarily targets CpGs in promoter regions, the HM450 expands coverage of exons, gene bodies, and 3′UTRs, targeting sites in 99% of RefSeq genes [13]. At each targeted position, the quantity of methylated (M) and unmethylated (U) DNA is measured by fluorescence intensity, and the proportion of methylated target is summarized by the average Beta value = M/(M + U), a value bounded by 0 and 1. Many such targets show skewed distributions near the boundary conditions, motivating the use of a Beta distribution for statistical modelling [5]. As not all targeted sites show variation in proportion of DNA methylation, our goal is to use non-specific filtering to reduce the dimension of variables in our analysis.

We refer to each targeted CpG site on the array as a feature, and evaluate a number of methods for ranking features and filtering for dimension reduction. A number of methods explicitly use parameters from, or variance-stabilizing transformations of, Beta distributions.

### Beta distribution

For a single feature, we model the distribution of DNA methylation across independent samples using a Beta distribution. Let X ~ Beta(α, β), $f\left(x\right)=\frac{1}{B\left(\alpha ,\beta \right)}x^{\alpha -1}{\left(1-x\right)}^{\beta -1}$, where $B\left(\alpha ,\beta \right)=\int_{0}^{1}u^{\alpha -1}{\left(1-u\right)}^{\beta -1}$ and α, β > 0. The mean and variance are given by $u=\frac{\alpha }{\alpha +\beta }$ and $\sigma^{2}=\frac{\mu \left(1-\mu \right)}{\left(\alpha +\beta +1\right)}$, respectively, with the variance a function of the mean. A useful reparameterization is *Beta*(*μ*, *ϕ*), with *ϕ* = *α* + *β* a precision parameter independent of the mean.

Transformations of the data can also lead to independence of the mean and variance. Du et al. (2010) [11] proposed the M-value, a (log2) logit transformation of the methylation proportion. However, the true variance stabilizing transformation for the Beta distribution is the cumulative distribution function (CDF), $Y=f_{B}\left(X;\alpha ,\beta \right)=\frac{1}{B\left(\alpha ,\beta \right)}\int_{0}^{X}t^{\alpha -1}{\left(1-t\right)}^{\beta -1}\mathrm{dt}$[12]. If the original data X follow a Beta distribution, the data after transformation (Y) will follow a Uniform distribution with mean 1/2 and variance 1/12. Any lack of fit of a single Beta distribution would suggest that the data arise from a mixture of Betas. We measure lack of fit using the distance of our transformed data from their expected distribution. Two filters below rank features using the CDF-transformed data, Y = CDF(X).

### Filter methods

We evaluate a total of eleven filters, eight based on ranking single statistics, and three based on a weighted score for combining ranks (Table 1). Filters 1 through 4 are commonly used methods today: Filter 1, standard deviation of Beta values (SD-b); Filter 2, standard deviation of M-values (logit-transformed Beta values) (SD-m); Filter 3, median absolute deviation of Beta values (MAD-b); Filter 4, DIP test, a measure of unimodality of Beta values (DIP) [14].

**Table 1:** Description of feature filtering methods

**Method**	**Description**	How to calculate the statistic*
SD-b	Standard deviation based on beta values	SD = sqrt(1/N ∑(Xi-mean(X))^2^)
SD-m	Standard deviation based on M values	SD = sqrt(1/N ∑(Xi-mean(X))^2^)
MAD	Median absolute deviation of beta values	median(|Xi-median(X)|)
DIP	Measure of unimodality in a sample	The max difference, over all sample points, between the empirical distribution function and the unimodal distribution function that minimizes that maximum difference
Precision	Inverse precision parameter	1/phi = 1/(mean(X)(1-mean(X))/SD^2^-1)
BQ-GOF	Beta Quantile Goodness-of-fit	Sum the absolute differences, over 25 quantile points, between the empirical distribution function and the expected beta distribution function
TM-GOF	Transformed Moment Goodness-of-fit	The Euclidean distance between the empirical standardized transformed moments and the expected center of the transformed moments (1/2,sqrt(1/12))
TQ-GOF	Transformed Quantile Goodness-of-fit	Sum the absolute differences, over 25 quantile points, between the empirical cumulative distribution function and the expected cumulative beta distribution function (uniform distribution function)
BR	Best rank of 8 single filter methods	The best rank value of 8 single filter methods (the highest rank value)
AR	Average of the top 2 ranks	The average of the best two rank values of 8 single filter methods
WAR	Weighted average of the top 4 ranks	The weighted average of the best four rank values of 8 single filter methods (weight = 4:3:2:1)

*Sample R code provided in Additional file 4.

Filters 5 through 8 are statistics that assume the data derive from a single Beta distribution. Filter 5, inverse precision (also known as adjusted SD) [15], ranks the data by an estimate of the (inverse) precision parameter, $1/\hat{\phi }=1/\left(\hat{\alpha }+\hat{\beta }\right)$, with alpha and beta estimated using method of moments estimators,

(1) $$\hat{\alpha }=\frac{{\bar{x}}^{2}\left(1-\bar{x}\right)-\bar{x}s^{2}}{s^{2}},\hat{\beta }=\frac{\left(1-\bar{x}\right)\left[\bar{x}\left(1-\bar{x}\right)-s^{2}\right]}{s^{2}},$$

where $\bar{x}$ and *s*^2^ are the mean and variance for a given feature. For this method the features with lower precision have higher variation. Filter 6, Beta-quantile Goodness-of-Fit (BQ-GOF), is a comparison of the observed to theoretical quantiles from a Beta distribution. It sums for each feature, the absolute difference between corresponding quantiles from the observed cumulative distribution function and the theoretical one obtained using the estimated parameters $\hat{\alpha },\hat{\beta }$.

Filters 7 and 8 measure goodness-of-fit on the CDF-transformed data, Y = CDF(X). Filter 7, Beta-Transformed Moments Goodness-of-Fit (TM-GOF). The CDF-transformed data are ranked using the distance of the mean, $\bar{y}$, and standard deviation, s_y_, from their expected values. The complete procedure is summarized as follows: 1. For each feature, estimate $\hat{\alpha },\hat{\beta }$; 2. Compute Y = CDF(X); 3. Compute, $\bar{y}$ and s_y_ the mean and standard deviation of the transformed data; 4. Calculate *s*_*y*_ and $s_{s_{y}}$, standard deviation for $\bar{y}$ and s_y_ across all features; 5. Rank features by their standardized Euclidean distance $\sqrt{{\left(\frac{\bar{y}-1/2}{s_{\bar{y}}}\right)}^{2}+{\left(\frac{s_{y}-\sqrt{1/12}}{s_{s_{y}}}\right)}^{2}}$. The features containing a mixture of Betas will have larger Euclidean distances compared to features that are from a single Beta distribution. Filter 8, Beta Transformed Quantiles Goodness-of-Fit (TQ-GOF), besides using the mean and SD of the CDF-transformed data as a pair of statistics to measure lack of fit, we can use the quantile differences between the observed CDF of Y and the theoretical CDF. Here, we rank the features by the sum of the absolute difference of the corresponding quantiles. This is similar to the BQ-GOF (Filter 6) except the quantiles, instead of the cumulative quantiles, are compared for the CDF-transformed data.

Filters 9 through 11 are summaries of the ranks from the individual statistics used above. Filter 9, Best Rank (BR) selects as the statistic the top rank across the eight statistics, Filter 10, Average Rank (AR), averages the top 2 ranks, and Filter 11, Weighted Average Rank (WAR), averages the top four ranks using weights 4:3:2:1, respectively.

### Simulation study

We perform a simulation study to evaluate the ability of the eleven non-specific filters to enrich a ranked list of features with those informative of subgroups. The data are simulated from distributions observed in our colon cancer data set (data set #1; see Real data sets below). In this data set, 6 out of 26 subjects (23%) contain a hypermethylation profile known as the CpG island methylator phenotype (CIMP), determined using a separate technology [10].

We simulate DNA methylation data from Beta distributions with parameters (μ_ij_,ϕ_ij_), where i = 1 or 2, for the CIMP and non-CIMP subsets, and j = 1,…,2000 indicates the feature. A random 10% of features are selected to be informative, with (μ_1j_,ϕ_1j_) and (μ_2j_,ϕ_2j_) estimated from the CIMP and non-CIMP subgroups, respectively. For the non-informative features, a single set of parameters (μ_.j_,ϕ_.j_) are estimated from the non-CIMP subgroup only, and used for both subgroups (i = 1,2). We simulate 200 samples, considering sample size ratios of 1:9, 1:1, and 9:1. As the feature characteristics vary between the two groups (e.g., CIMP cancers shows higher mean and variance of measures on average compared to non-CIMP cancers, Additional file 1: Figure S1A-C), the ratios 1:9 and 9:1 can represent very different scenarios. For 100 replicate data sets, we rank the 2000 features based on the different filter methods. For each data set, the same 1800 distributions are used for the non-informative features, and a new random sample of 200 features is selected for the informative features. For each data set and each filter statistic, we rank the features by the statistic, and compute sensitivity and specificity for identifying the 200 differentially methylated features, for feature lists of all possible lengths. The average sensitivity and specificity is computed over the 100 replicate data sets for each filter, and presented using receiver operating characteristic (ROC) curves.

In addition, we compare for the different filtering methods the sample misclassification rates when performing cluster analysis using a Recursive-Partitioning Mixture Model (RPMM) [5]. RPMM was designed for clustering DNA methylation signatures, and clusters samples using a mixture of Beta distributions in a recursive partitioning routine. For each filter method, cluster analysis of the samples is performed on the top 100, 200, and 400 ranked features (5%, 10%, 20%, respectively), when the true percentage of informative probes was 10% of all features. In the results we will see that all filter methods performed well when the distributions of the informative features mimicked the distributions observed in the real data set. We attributed this to a very strong cluster signal from the subset of informative features. In an attempt to differentiate the performance of the filter methods, we restricted the distributions of the informative features to those from a subset of features showing a smaller effect size. We defined the effect size of a feature by $\hat{\theta }=\ln\frac{{\hat{\mu }}_{2j}/\left(1-{\hat{\mu }}_{2j}\right)}{{\hat{\mu }}_{1j}/\left(1-{\hat{\mu }}_{1j}\right)}$, and sampled 200 informative features from the subset with $\left|\hat{\theta }\right|\leq 1$, or $\left|\hat{\theta }\right|\leq 0.5$. The 1800 non-informative features remain unchanged from the earlier ROC-curve evaluation. The limit on the effect size of the informative features reduced the cluster signal in the data, and resulted in greater variation in performance among the different filtering approaches.

### Real data sets

Finally, we apply our filtering methods to eight Illumina data sets. The data sets were selected to span a variety of biological conditions and include data generated on the HM450 and HM27 platform. We selected three cancer-only, four tumor-normal tissue, and one normal blood data set. The three cancer-only data sets include one colon and two glioblastoma, cancer types known to have a distinct subtype defined by the CpG island methylator phenotype (CIMP). The four tumor-normal tissue data sets include two kidney and two breast data sets. The normal blood data set is selected to evaluate the filter methods in the presence of the strong quantitative risk factor, age. Further details are provided in Table 2 and Supplemental Material (Additional files 2 and 3). All of the data are anonymized, and this study did not require institutional review board approval.

**Table 2:** Description of data sets used in application analysis

**Data set**	**Description**	**Platform**	**Source**	**# of probes after preprocessing**	**# of samples**
1	Colon cancer	HM27	Local	19,965	20 NONCIMP vs. 6 CIMP
2	Glioblastoma	HM27	TCGA, plate 1,2,3,10	20,549	74 NONCIMP vs. 12 CIMP
3	Glioblastoma	HM450	TCGA, plate 79,111,130	374,601	93 NONCIMP vs. 6 CIMP
4	Kidney	HM27	TCGA, plate 64	21,624	50 KIRC vs. 45 normal
5	Kidney	HM450	TCGA, all KIRC	374,708	283 KIRC vs.160 normal
6	Breast	HM27	TCGA, plate 93	21,787	37 Infiltrating Ductal Carcinoma vs. 20 normal
7	Breast	HM450	TCGA, plate 109	377,853	56 Infiltrating Ductal Carcinoma vs. 17 normal
8	Normal blood	HM450	GEO:GSE40279, plate 2	383,911	84 blood samples

We perform RPMM cluster analysis on different lists of filtered features to assess the ability of different filtering approaches to identify (1) cancer subtypes, (2) cancer/normal tissue types, or (3) young from old individuals. For each data set we applied the 11 filtering methods, each time selecting the top 1000 features for RPMM clustering. We also considered a hybrid variable selection approach, in which we pool the top 500 features selected from two filters methods above (SD-b and TM-GOF). These filters are chosen because they each perform well for different biological conditions studied (see Results below). For data sets 1–7 the misclassification rate was computed by comparing the top two clusters to the known tissue types. We also evaluated the cluster agreement between the clusters identified by the RPMM routine (typically varying from 2–6 groups) with our two known tissue classes using the adjusted rand index. For data set 8, we evaluated the differences in mean age between the two major cluster groups. To evaluate the general effect of filtering the data, we analyzed the HM27 data sets without any filtering, and the HM450 data sets after selecting a random subset of 1000 features. Feature reduction for the HM450 data was necessary, as the cluster analysis software required a lower dimensional data set in order to run. Also, since the HM450 clustering results varied with random selection of features, we repeated the analysis ten times and report the average misclassification error rate over the ten replicates (data sets 1–7).

For two filter methods that show good performance (SD-b and TM-GOF, filters #1 and #7), we report the frequency of the top selected features by genomic context. On the HM27 array the selected probes are characterized using the UCSC definition of CpG island and the gene-based definitions provided by Illumina: “promoter”, “transcribed region”, “exonic region”, and “intronic region”. For the HM450 array we stratified four gene-based categories (Promoter/Exon/Intron/Intergenic) [16] by their position relative to a CpG island (hg19 UCSC definition).

All analyses are performed using the R programming language 2.15.2 (http://www.r-project.org). Infinium data were processed using the methylumi package in Bioconductor, using a combination of Normal-Exponential background correction, dye bias equalization, and beta-mixture quantile normalization (BMIQ) to remove technical artifacts [17,18]. With a goal of discovering latent disease subtypes, we removed features occurring on the X and Y chromosomes which would be enriched for sex-related variation, and features with other data quality issues (e.g. contain common SNP within 10 bp of the target CpG that may misrepresent DNA methylation level, or map to multiple regions of genome hg19 and lack target specificity. Common SNPs are defined as having MAF > 0.01 in dbSNP build 135 per the UCSC snp135common track.) After pre-filtering, 22,198 CpG targets remain on the HM27 array, and 384,310 on the HM450. Sample R code for the filtering methods is provided in Additional file 4.

## Results

### Colon cancer data

**Figure 1:**
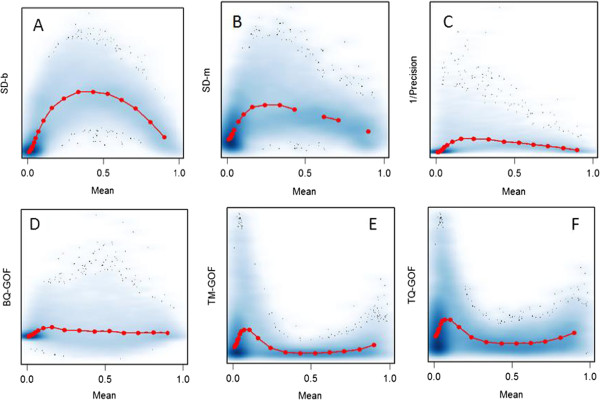
**Smoothed scatter plots showing six filter statistics vs. the mean DNA methylation (Beta) value (22198 features, 26 colon cancer samples). A**. SD-b: standard deviation of Beta values; **B**. SD-m: standard deviation of M-values; **C**. 1/Precision: inverse of precision parameter; **D**. BQ-GOF: Beta Quantile Goodness-Of-Fit; **E**. TM-GOF: Transformed Moment Goodness-Of-Fit; **F**. TQ-GOF: Transformed Quantile Goodness-Of-Fit. Red line in each figure indicates the median statistic values.

Figure 1 shows the relationships between six filter statistics and mean DNA methylation level in a study of 26 colon cancer tissues. In this collection of heterogeneous cancers (23% CIMP and 77% non-CIMP), we see a strong relationship between standard deviation and the mean value (Figure 1A), and selecting features with high variation (SD-b) biases the selection to those with mean near 0.5. This relationship is reduced for alternate filter statistics (Figure 1B-1E). Therefore, depending on the filter statistic employed, a different set of top ranked features may be retained for statistical evaluation.

### Simulation study

#### Enrichment of ranked lists

**Figure 2:**
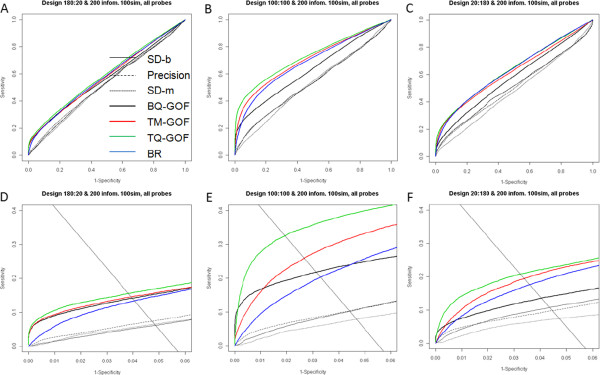
**ROC curves for 7 filtering methods (2 groups, 200 informative features out of 2000, 200 samples, 100 simulated data sets).** For each data set the sensitivity and specificity of selecting informative features using the top ranked list (1**–**2000 features) are averaged over 100 replications. Figure **A-C** show ROC curves for 7 listed filtering methods: SD-b, Precision, SD-m, BQ-GOF, TM-GOF, TQ-GOF, and BR (best rank) under different sample ratio scenarios: A. Sample size ratio 9:1 (non-CIMP/CIMP); B. Sample size ratio 1:1; **C**. Sample size ratio 1:9. The bottom three panels D-F are partial ROC curves obtained from the panels **A-C** by restricting the axis ranges to the region relevant to the diagonal line. The solid black diagonal line in Figure **D-F** indicates the estimated sensitivity and specificity levels for a list of 100 genes.

The ROC curves from the analysis of simulated data show the ability of the filters to enrich the top ranking genes with those truly informative of subgroup (Figure 2). The features that enrich the top of the list will show increasing sensitivity under low false-positives, falling above the diagonal line, and having an area under the curve (AUC) greater than 0.5. Figure 2 shows that for all scenarios considered, filters that combine a Beta variance stabilization transformation with goodness-of-fit statistic (TQ-GOF, TM-GOF, BQ-GOF) appear to enrich the most highly ranked features with ones informative for cluster subgroup. The filters SD-b, SD-m, and Precision appear non-informative, with 95% confidence intervals for the AUC containing 0.5 (Additional file 5: Table S1). The figures also show that for the informative filters, the greatest enrichment occurs when the subgroups have equal sample size. This is to be expected, as equal sample sizes will give the greatest power to detect differential DNA methylation in supervised analyses. Between the two analyses with unequal sample sizes, the better discrimination occurs when the group with larger variance has the larger sample size (Figure 2C, F).In ROC analyses, another quantity of interest is the partial AUC, the AUC for a given false-positive rate. In this setting, fixing the error rate will give a variable number of features for different filters. Instead, we select a fixed number of features (p) to discuss the sensitivity and specificity. This approach reflects how non-specific filtering is performed in practice. The solid black diagonal line in Figure 2D-F indicates the estimated sensitivity and specificity levels for the top 100 features. The diagonal line connects the boundary points indicating the maximum true-positive fraction (y-axis) for 0 false-positives (x-axis) and the maximum false-positive fraction for 0 true-positives. In the simulation, the true number of informative features is always 200 out of 2000. Thus, the maximum possible true-positive fraction is 0.5, corresponding to all 100 features selected being true positives (100/200 true-positives and 0/1800 false-positives); the maximum possible false-positive fraction is 0.056 (100/1800 false-positives and 0/200 true positives). The diagonal lines in Figures 2D-F connect the coordinates for these boundary points: (0,0.5) and (0.056,0), respectively. We estimate the sensitivity and specificity for the top ranked 100 features from each filter by the coordinates where the ROC curve crosses the diagonal line. These intersection points provide a more clear comparison of enrichment for the top ranking features than is evident when viewing the entire ROC curve. For subgroups of equal size, the TQ-GOF statistic shows the greatest sensitivity and specificity in selecting informative features for a fixed number of features. For unequal sized subgroups, the methods TM-GOF and BQ-GOF, performed competitively.

#### Cluster analysis

**Figure 3:**
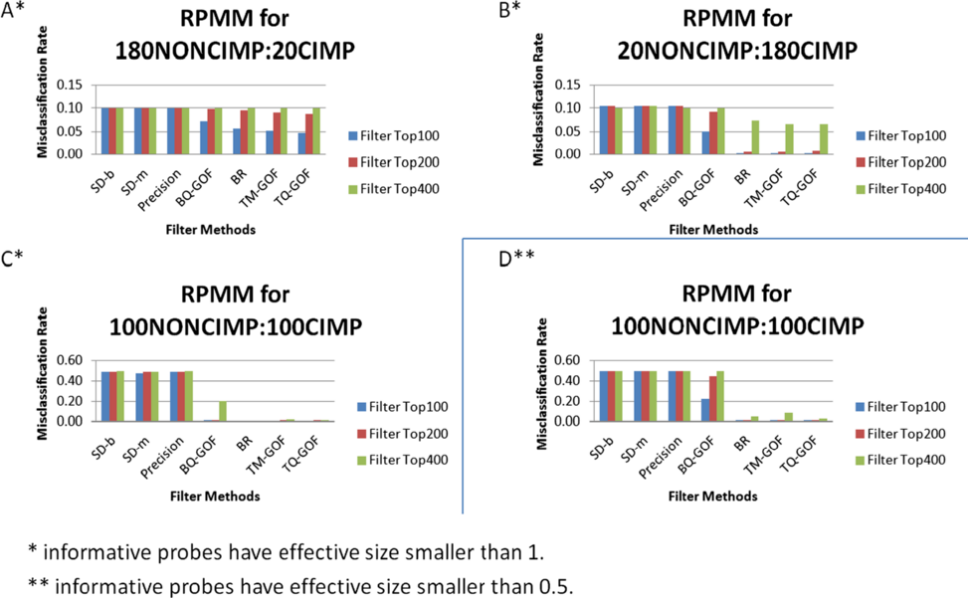
**Misclassification rates of RPMM cluster analysis using top filtered features for 7 filtering methods (2 groups, 200 informative features out of 2000, 200 samples, 100 simulated data sets).** Average 100 simulations of misclassification rates from a cluster analysis performed using RPMM, for the top 100, 200, or 400 features of seven different filtering methods under different sample size ratios. A*. Sample size ratio 9:1 (non-CIMP/CIMP); B*. Sample size ratio 1:9; C* &D**. Sample size ratio 1:1. For A*-C*: informative features have effective size smaller than 1; For D**: informative features have effective size smaller than 0.5.

Applying cluster analysis to the data simulated in Figure 2, all methods performed nearly perfectly (results not shown). Presumably this is due to the selection of a few features with very large signal between the two cancer subtypes. To introduce variation in behaviour, we reduced the effect sizes for the informative features in the simulation (see Methods). Figure 3 shows the misclassification error rates from a cluster analysis of data simulated under these reduced effect sizes. For all scenarios, TM-GOF and TQ-GOF performed best among all single statistic methods. We see that the Precision filter, SD-b and SD-m performed worst. Filters MAD and DIP also performed poorly (data not shown). Regardless of the number of features retained, the Precision filter was unable to find the correct subgroups in the cluster analysis. For other methods, the misclassification rates increased as we increased the number of features in the analysis. Among the three summary methods, BR and AR performed similarly to the best single filter methods (AR data not shown); the WAR filter did not perform as well (data not shown). These results are consistent with previous ROC curves (Figure 2).We note that the maximum error rate for each panel in Figure 3 depended on the sample sizes in the two subgroups. When there are no clear clusters in the data, RPMM tends to find one big cluster. This resulted in a maximum error rate of 10% (=20/200) for sample size ratios of 1:9 and 9:1 (Figure 3A and B) and an error rate of 50% (=100/200) when the sample sizes were equal (Figure 3C and D).

### Real data application

Table 3 shows the misclassification rates of RPMM cluster analysis after variable filtering on a variety of cancer data sets. Error rates are also reported in the absence of filtering, or after random feature selection. For the colon and glioblastoma cancer data sets containing CIMP and non-CIMP cancer subtypes, (the colon data set being the one our simulation study mimicked), TM-GOF and TQ-GOF consistently showed low misclassification rates, as expected from our simulation study. For the data sets having both cancer and non-cancer tissues, these same filters performed much worse than other methods (Table 3). For the kidney samples (data sets #4-5), all methods *except* TM-GOF and TQ-GOF performed well, including no filtering for the HM27 data and random filtering for the HM450. Interestingly, for the breast tissues (data sets #6 and #7), SD-b and SD-m performed best. None of the summary-based filter methods ever performed better than the best single-filter method. They also performed inconsistently across the different data sets. The hybrid approach that combined an equal number of top SD-b probes with TM-GOF probes showed some merit. Sometimes the hybrid selection scheme behaved as well as the best single filter method (Colon data set #1, Kidney data sets #4, #5), and at other times it resulted in error rates that were intermediate between the other two (Glioblastoma data set #3, Breast cancer data sets #6, #7). Overall, the TM-GOF and TQ-GOF methods consistently performed best for identifying the CIMP subgroup in cancer data, while the SD-b filter performed best at distinguishing cancer from non-cancer tissue.

**Table 3:** Misclassification rate of RPMM cluster analysis to find 2 groups using different variable filtering methods (top 1000 features)

	**Data set 1**	**Data set 2**	**Data set 3**	**Data set 4**	**Data set 5**	**Data set 6**	**Data set 7**
**Tissue type**	Colon cancer	Glioblastoma	Glioblastoma	Kidney	Kidney	Breast	Breast
**Platform**	HM27	HM27	HM450	HM27	HM450	HM27	HM450
**# of samples**	20 non-CIMP vs. 6 CIMP	74 non-CIMP vs. 12 CIMP	93 non-CIMP vs. 6 CIMP	50 KIRC vs. 45 non-cancer	283 KIRC vs. 160 non-cancer	37 Breast cancer vs. 20 non-cancer	56 Breast cancer vs. 17 non-cancer
**No filter**	0.31	0.22	NA	0	NA	0.12	NA
**Filter top 1000 by:**							
Random *	0.34	0.27	0.40	0.004	0.005	0.12	0.20
**SD-b**	0.19	0.07	0.49	0	0.02	0	0.12
**SD-m**	0.12	0.07	0.42	0.02	0.03	0.12	0.08
**MAD**	0.38	0.35	0.49	0	0.005	0	0.14
**DIP**	0.23	0.36	0.45	0	0.005	0	0.14
**Precision**	0.08	0	0.10	0.03	0.01	0.11	0.22
**BQ-GOF**	0.19	0	0.07	0	0.01	0.25	0.23
**TM-GOF**	0.08	0.02	0.06	0.36	0.47	0.44	0.49
**TQ-GOF**	0.08	0.03	0.06	0.35	0.47	0.44	0.48
**BR**	0.12	0.02	0.11	0.02	0.02	0.23	0.19
**AR**	0.08	0.06	0.11	0.02	0.02	0.25	0.19
**WAR**	0.12	0.07	0.45	0.02	0.01	0.11	0.10
SD-b + TM-GOF**	0.08	0.07	0.20	0.05	0.01	0.26	0.36

NA = not applicable; Too many features for RPMM to run.

*Average from 10 analyses of randomly sampled feature sets.

**Combine top 500 SD-b + top 500 TM-GOF features.

**Figure 4:**
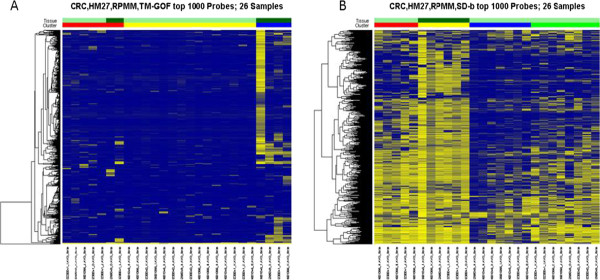
**Heatmaps of RPMM cluster analysis using top 1000 filtered features by A) TM-GOF or B) SD-b methods using 26 colon cancer samples (data set #1).** Rows represent features and columns represent samples; yellow represents high DNA methylation and blue represents low. The color bars at the top of the columns indicate sample tissue types (row 1) and clusters (row 2). In row 1 dark and light green indicate CIMP and non-CIMP tumors, respectively. In row 2 red, yellow, blue and green bars indicate the sample clusters found after two divisions of clustering using RPMM. In Figure **A**, the red and yellow clusters are identified at the second division, and no subdivision of the blue cluster is found. In Figure **B**, the red and yellow clusters separate in the second division, as do the blue and green clusters.

**Figure 5:**
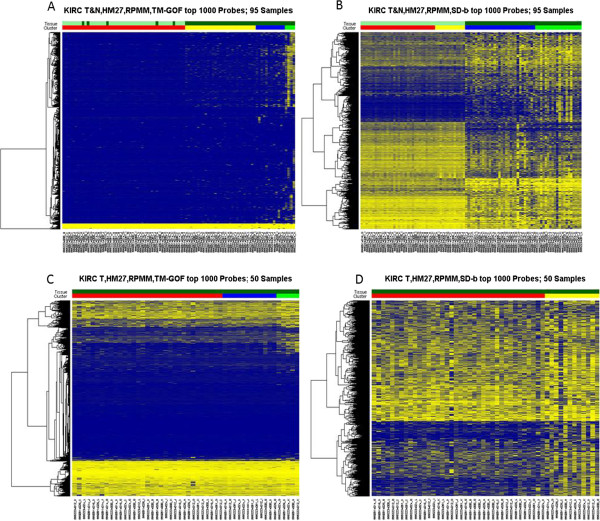
**Heatmaps of RPMM cluster analysis using top 1000 filtered features by TM-GOF (A,C) or SD-b (B,D) methods using 95 kidney cancer-non-cancer samples (data set #4).** Rows represent features and columns represent samples; yellow represents high DNA methylation and blue represents low. The color bars at the top of the columns indicate sample tissue types (row 1) and clusters (row 2). In row 1 dark and light green indicate cancer and non-cancer samples, respectively. In row 2 red, yellow, blue and green bars indicate the sample clusters found after two divisions of clustering using RPMM. Figure **A** &**B** show heatmaps of all 95 kidney samples using top 1000 features filtered by TM-GOF or by SD-b method, respectively. Figures **C** &**D** show heatmaps of 50 kidney tumors using top 1000 features filtered by TM-GOF or by SD-b method, respectively. In **C**, the blue and green bar clusters are found at the second separation.

To visualize the different performance of our top filters, TM-GOF and SD-b, we created heatmaps of the top 1000 filtered features following RPMM clustering. Figures 4 and 5 show the clusters identified for the colon cancer data set (data set #1, tumor-only) and the TCGA kidney data set (data set #4, tumor and non-tumor kidney), respectively. Using TM-GOF in the colon cancer data, our subcluster identified 4 out of 6 CIMP samples, leaving 2 CIMP samples misclassified in our first split of the data (Figure 4A). (We note that we use the word “misclassified” loosely, as our definition of CIMP is likely not a gold standard (see Additional file 2, Data set #1)). Using SD-b as the filter, the first split identified two clusters more equal in sample size (11 and 15), misclassifying 5 non-CIMP samples (Figure 4B). Interestingly, in the next split of the data all 6 CIMP samples separated themselves from the others, appearing together in one of the four sub clusters (Figure 4B). Thus the CIMP subtype was found by SD-b filtering, but only after further sub-clustering. Using the adjusted rand index measure of the co-clustering of sample pairs by cluster category and tissue label the TM-GOF filter showed superiority over the SD-b filter because of the smaller number of clusters estimated by the clustering method (Additional file 6: Table S2). In Figures 4A and B, 47 out of 1000 features are shared by the two filter methods. For data set #4 (Figure 5), SD-b filter resulted in the successful identification of tumor and normal kidney samples at the first division (Figure 5B). Using TM-GOF, the first cluster division identified a subtype of 4 cancer samples (Figure 5A, light green) with high DNA methylation in a subset of features. However, a second division of the data resulted in the separation of non-cancer tissues from the cancer samples (red bar). Thus, again we found the substructure sought in the cluster analysis, but not until the second division of the clusters. In Figure 5A and B, only 4 of the 1000 features overlapped. Interestingly, using different feature selection methods the cluster substructure became similar for both data sets, even when the first split found different subgroups. This common substructure in the data set was captured by the adjusted rand index (Additional file 6: Table S2). However, this same cluster substructure was not reproduced on the HM450 platform.We also asked whether omitting features with outlier values might discover larger clusters than the small disease subset discovered when filtering using TM-GOF (Figure 5A). We omitted all features with values greater than or less than the median Beta value +/−3 times the inter quartile range (IQR), excluding a relatively large number of features from the kidney data set (data set #4). We then selected the top 1000 features using TM-GOF, performed cluster analysis, and found perfect discrimination of cancer and non-cancer kidney (Figure not shown). This confirmed that the TM-GOF filter favoured features that identified disease subtypes prior to its selection of features identifying tissue disease state.

This same removal of features with outliers, followed by filtering the top 1000 features and cluster analysis, was performed on data sets #5-#7. Each time we found TM-GOF was able to find disease state clusters at the first division just as well as SD-b (results not shown). However, for the CIMP cancer data sets (data sets #1-3), a pre-filtering of features with outliers (using median+/−3*IQR criteria) resulted in equally bad clustering for all filtering methods (data not shown). These results suggest that broad pre-filtering using this definition removed features informative for a CIMP classification.Next we asked if we could find the same tumor substructure in the kidney data set if we had started with the 50 kidney tumors only. In general, this appeared to be the case. Using TM-GOF as our filter, we identified a distinct subgroup of five tumors, four of which were those previously identified when clustering the larger set of tumor and non-tumor tissue (Figure 5C). A subset of 50 features (5%) is shared by the two analyses, selected in the top 1000 features of both data sets (Figure 5A and C). When using features selected using SD-b, the tumors are separated into subgroups of size 12 and 38 (Figure 5D). The cluster of five tumors identified from the TM-GOF filter is a subset of the cluster of 12 identified using SD-b filter; a subset of 44 of the 1000 features (4.4%) were selected by both filter methods (Figure 5C and D).

The last data set analyzed was whole blood (data set #8), selected to compare the different filter methods when there is structure due to age, a continuous variable. Table 4 summarizes the mean age in the top two clusters after filtering the top 1000 features and performing RPMM in 84 whole blood samples. The number of samples in each cluster varied considerably depending on the filtering method. Selecting features ranked by SD-b resulted in two groups approximately similar in size (52 vs 32) whereas selecting features ranked by MAD resulted in the detection of only a single cluster (n = 84) (Table 4). The difference in mean age between the top two clusters was greatest when using the hybrid SD-b and TM-GOF filtering method (difference = 7.3 years, p = 0.02) or WAR (difference = 7.2 years, p = 0.01), and the filters SD-b, SD-m, and AR all recovered differences in mean age around 6.5 years (p = 0.03-0.04). Two filters using the Beta-distribution variance stabilizing transformation (TM-GOF and TQ-GOF) tended to find groups most unequal in sample size, but not statistically significantly associated with age (p > 0.05). Interestingly, the Precision filter found groups with nearly balanced sample size, but did not discriminate samples by age (difference = 3.5, p = 0.25).

**Table 4:** Mean age in two clusters identified by RPMM using different filtering methods on blood samples of a normal population

**Filter method**	**Sample size group 1**	**Sample size group 2**	**Mean age group 1**	**Mean age group 2**	**Difference in mean ages**	**T test p-value**
**MAD**	84	0	59.54	0	NA	NA
**DIP**	84	0	59.54	0	NA	NA
**SD-b**	52	32	57.10	63.50	6.40	0.03
**SD-m**	64	20	57.92	64.70	6.78	0.04
**Precision**	40	44	57.73	61.18	3.46	0.25
**BQ-GOF**	62	22	58.21	63.27	5.06	0.09
**TM-GOF**	75	9	59.11	63.11	4.00	0.38
**TQ-GOF**	75	9	59.11	63.11	4.00	0.38
**BR**	57	27	57.89	63.00	5.11	0.08
**AR**	56	28	57.38	63.86	6.48	0.03
**WAR**	55	29	57.04	64.28	7.24	0.01
SD-b + TM-GOF*	56	28	57.09	64.43	7.34	0.02

*Combine top 500 SD-b + top 500 TM-GOF features.

We report the genomic context of the features selected by TM-GOF and SD-b, our two best performing filter methods (Additional file 7: Table S3a and 3b). For the colon cancer and glioblastoma data sets (#1-3), both filter methods enriched for features in CpG islands, which makes sense for detecting a CpG island methylator phenotype cancer subtype. In the cancer versus normal tissue comparisons, the different filters prioritized different feature subsets. For the HM27 data, SD-b prioritized features from non-CpG island regions while TM-GOF still prioritized features from CpG island regions. Both filters enriched for features from exonic regions, with only SD-b from the Kidney data set (#4) preferentially selecting features in promoters. For the HM450 Kidney cancer vs non-cancer tissue comparison, both filter methods over-represented non-CpG island features, however TM-GOF selected more from exons and SD-b selected more from introns and intergenic regions. The Breast cancer versus non-cancer tissue (data set #7) showed the greatest variation in enrichment categories by the TM-GOF and SD-b filter methods. The better performing SD-b filter selected intergenic features, both inside and outside CpG islands. In general, across all HM450 data sets the TM-GOF filter selected more features from CpG island promoters than the SD-b filter (Additional file 7: Table S3b). At the same time, the SD-b filter selected more features from non-CpG island intergenic regions. Thus the two filters were sensitive to prioritizing features in different regions of the genome. This likely explains their different performance for clustering samples from different biological conditions.

We comment briefly on the effect processing HM450 data has on feature selection. We present results for data processed using a combination of background correction, dye-bias [17] and BMIQ normalization [18]. We performed analyses both with and without BMIQ normalization and saw a huge enrichment of design type 1 features prior to BMIQ normalization. Following BMIQ normalization the distribution of selected design type 1 and type 2 features aligned more closely to the distribution on the array. Interestingly, despite the different probe types being selected after normalization, the distribution of features by genomic context varied little (results not shown). Thus we believe the genomic context of the feature is a stronger predictor of feature selection than the platform feature design type.

## Discussion

We used both simulated and real data to evaluate the performance of a number of variable filtering methods for cluster analysis, when the variables are proportions that are bounded on the 0 to 1 scale. Both the simulated and real data show that TM-GOF and TQ-GOF are the best at identifying a subset of cancers having the CpG island methylator phenotype (CIMP). The new filters that use a Beta variance stabilizing transformation are very sensitive to outlier measurements. This may benefit the search for low frequency cancer subtypes that have extreme values occurring across a large number of features (e.g. CpG island methylation phenotype), but may not translate to an ability to directly cluster cancer versus normal tissues well. For clustering cancer versus normal tissue, an outlier removal step was required before the tissue clusters could be properly recovered. In general, the cancer-based simulation study results were not generalizable to the clustering of normal tissue, or tumor versus non-tumor tissue, suggesting that the filter methods are sensitive to the variation in observed DNA methylation distributions due to the underlying biology.

Overall, SD-b performed very well in the real data examples including normal tissues. One explanation could be that the SD-b filter enriches for features in regions having cell-type specific DNA methylation differences. It tended to find groups of approximately equal size, finding a separation of groups by mean age for the normal blood samples that was statistically significant (difference = 6.4 years, p = 0.03); in the cancer and non-cancer studies it identified clusters based on disease state in the first partitioning of samples. Although at first glance it appeared to perform poorly in detecting the CIMP subtype in the colon cancer data, upon further partitioning of the data the sub-cluster of interest appeared. It is unknown if this is a coincidence from the data selected in this study. Although the SD-b filter did not show a high AUC in the simulation study, it did cluster the samples perfectly using RPMM when we did not set boundaries on the largest effect size, suggesting that the cluster analysis can be strongly influenced by a small number of highly informative features.

We found the SD-b and TM-GOF filters tended to prioritize features in different areas of the genome. For each HM450 data set, TM-GOF selected a higher number of features in CGI promoters compared to SD-b, while SD-b selected a larger number of features in non-CGI intergenic regions. The better performance of TM-GOF for detecting a CIMP subtype in cancer, and SD-b for clustering normal tissues, suggests that features in different regions of the genome are not equally informative for all biological conditions. A recent study reported a novel classification of breast cancer using markers of normal-breast epithelial cell subtypes [19], and might explain our superior classification of cancer versus normal breast using SD-b, the filter performing best in normal tissues.

The SD-m filter was a close competitor to SD-b, but Precision showed an unexplained sensitivity to tissue type. Both Precision and SD-m performed slightly better than SD-b in the analysis of CIMP cancers and nearly as well in clustering cancer and non-cancer kidney and breast. In the analysis of whole blood, SD-m found clusters that correlated with age, but Precision did not. Because of this unexplained sensitivity of Precision to non-cancer tissue we do not recommend its general use.

One filter method not included in our comparison is arcsine-square-root transformation, also a good variance stabilization method suitable for data bounded between 0 and 1. Similar to the logit transformation, the arcsine-square-root transformation can be written as an incomplete beta function having only one parameter [12]. Thus we would expect a filter based on its standard deviation to behave similarly to our filter using the logit transformed data (SD-m). Another statistic that could be used as a filter method is the SD ratio, $\mathrm{SD}\left(X\right)/\sqrt{\mathrm{mean}\left(X\right)\left(1-\mathrm{mean}\left(X\right)\right)}$. For a Beta distributed variable X, this ratio is equivalent to the (inverse) precision method used in this paper (filter #5).

Although the summary-based filtering methods take advantage of using the top ranked filter methods, they are not always more robust than the single-filter methods. This is because sometimes the best rank of a feature can be affected by a single non-informative filtering method. Thus, due to the different (and somewhat complimentary) characteristics of the features enriched by SD-b and TM-GOF methods, we prefer to use both SD-b and TM-GOF methods for any data when our main purpose of cluster analysis is to identify novel subgroups. Our results suggest that SD-b is very robust in enriching for features that identify big subgroups, while TM-GOF and TQ-GOF are very sensitive in enriching features to identify low frequency cancer subtypes that have outlying values occurring across a large number of features (e.g. CpG island methylation phenotype).

We noticed that in the data sets comparing DNA methylation in cancer to non-cancer tissue, the differences in standard deviation (SD) between sample groups are not symmetrically distributed. The majority of features have a much higher SD in cancer samples than in normal samples. However, in data sets with non-CIMP vs. CIMP cancer subtypes, the differences in SD are symmetrically distributed with mean around zero (data not shown). This suggests that SD on beta values may be more informative in data sets with huge SD differences between subgroups than in data sets with balanced SD differences around zero.

One limitation of our simulation design is that for each tissue subgroup our measures are simulated to follow a beta distribution and the best performing filter methods make proper use of this knowledge. In reality, a mixture of betas might yield a more realistic measure from a population of mixed cell types. However, instead of simulating this added complexity which would require additional model assumptions, we chose to look for patterns of behaviour from the analysis of a variety of real data sets that spanned different biological conditions (e.g. tumor only, tumor versus normal, or single normal tissue). This evaluation shows: (1) the top two filter methods, TM-GOF and SD-b, prioritize features from different parts of the genome, (2) TM-GOF is much more susceptible to outlier measures, and (3) that the underlying biology can drive their performance.

One question not addressed in this study is the number of features to carry forward to the cluster analysis. One might plot the filter statistics to see if they show a bimodal distribution, suggesting subgroups of features with different behaviour. In our experience, the statistics are unimodal so we tend to use a number of thresholds for features selection (e.g. top 1000, top 2000, top 5000 features), and evaluate the robustness of our groups across the different feature lists.

## Conclusions

We found two filter statistics, SD-b and TM-GOF, outperform the rest in different data sets with different nature. We would suggest using each one, as cluster analysis is for the purpose of class discovery and the two methods tend to prioritize different sets of features, thus serving as complimentary feature enrichment methods for DNA methylation data.

### Availability of supporting data

The Cancer Genome Atlas data (data sets 2–7) are publicly available from the TCGA data portal (https://tcga-data.nci.nih.gov/tcga/). The blood samples (data set 8) are the subset of samples from plate 2 of GEO data set GSE40279 (http://www.ncbi.nlm.nih.gov/geo/query/acc.cgi?acc=GSE40279). The exact samples included in our analysis are provided in Additional file 3.

## Competing interests

The authors declare that they have no competing interests.

## Authors’ contributions

XW performed the analyses, PWL, SG and KDS served as advisors. TH created the HumanMethylation450 genome annotations. The manuscript was written by XW and KDS. All authors read and approved the final manuscript.

## Supplementary Material

Additional file 4 Sample R code to compute non-specific filter methods. Click here for file

Additional file 1: Figure S1 Distribution of statistics in Colon Cancer data set (data set #1). Click here for file

Additional file 2 Real data sets. Click here for file

Additional file 3 Sample ids included in data sets 2 through 8. Click here for file

Additional file 5: Table S1 Area under the curve (95% confidence interval) for simulation results in Figure 2. Click here for file

Additional file 6: Table S2 Adjusted Rand Index of RPMM cluster analysis result using a variety of filtering methods for multiple data sets. Click here for file

Additional file 7: Table S3 Genomic context of the features selected by the top two filter methods. **a.** For HM27 platform (N/%). **b.** For HM450 platform (N/%). Click here for file
