# Supplementary material for: Non-specific filtering of beta-distributed data
Source: BMC Bioinformatics. 2014 Jun 19;15:199. doi: 10.1186/1471-2105-15-199 (PMC4230495; doi:10.1186/1471-2105-15-199)
Supplement: Additional file 1: Figure S1 — Distribution of statistics in Colon Cancer data set (data set #1). [file 1471-2105-15-199-S1.docx]

**Additional file 1 – Supplemental Figure**

**Supplemental Figure. Distribution of statistics in Colon Cancer data set (data set #1).**


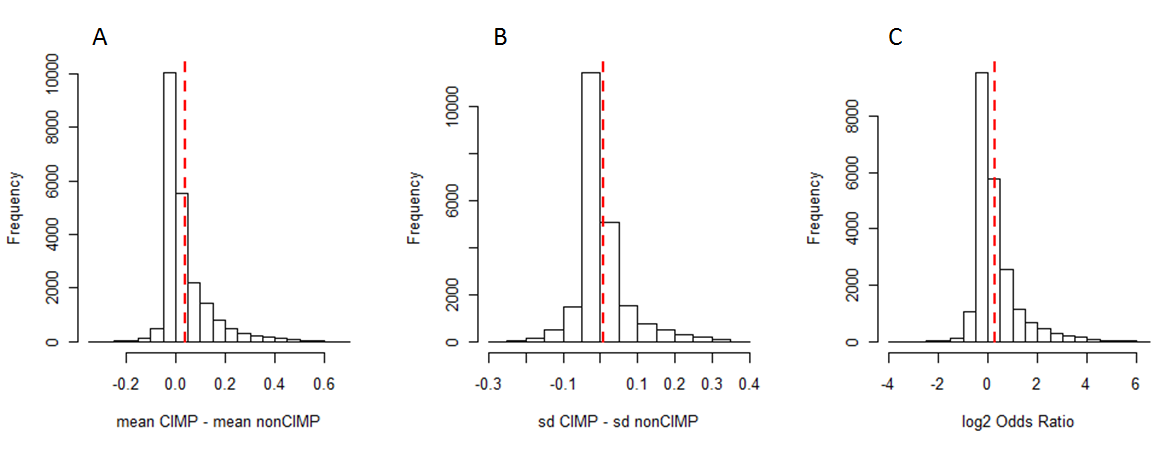


**Supplemental Figure 1.** The distribution of statistics between CIMP and non-CIMP Colon cancer samples for 22198 features. A. The histogram of mean differences between CIMP and non-CIMP samples for 22198 features. B. The histogram of standard deviation differences between CIMP and non-CIMP samples for 22198 features. C. The histogram of log2(Odds Ratio) for 22198 features. The log2(OR) is calculated using, where $\hat{\mu}_{1j}$ is the mean for non-CIMP tumors, and $\hat{\mu}_{2j}$ is the mean for CIMP tumors. The red vertical line indicates mean.
