# Supplementary material for: Non-specific filtering of beta-distributed data
Source: BMC Bioinformatics. 2014 Jun 19;15:199. doi: 10.1186/1471-2105-15-199 (PMC4230495; doi:10.1186/1471-2105-15-199)
Supplement: Additional file 2 — Real data sets. [file 1471-2105-15-199-S2.docx]

# Additional file 2 - Real data sets

Table 2 describes eight data sets used to evaluate the different filtering methods in conjunction with RPMM cluster analysis. As RPMM requires complete data for all features, we omitted features having any missing beta values as determined by the detection p-value. Features were also filtered based on quality control criteria listed in the methods section (e.g. SNP at target CpG or nearest 10bps in feature body, 15-bp repetitive elements, etc.). The data sets and total number of features after filtering are listed below.

Data set #1 is HM27 data from 26 colon cancers generated at the USC Epigenome Center. A previous analysis of these samples using the MethyLight technology found a subset of 6 cancers to have the CpG island methylator phenotype (CIMP). This independent assessment of CIMP subtype is used to assess our clustering results. Parameter values in our simulation study were sampled from features in this data set (N=19,965 features).

Data sets #2 to #7 are downloaded from The Cancer Genome Atlas (TCGA) data portal. We downloaded level 1 data and performed our own data processing as described in the methods section.

Data set #2 is HM27 data (plates 1, 2, 3, and 10) of 86 glioblastoma cancers (GBM). A hierarchical cluster analysis on 1362 out of the 1503 CIMP-related features published by Noushmehr et al. (Noushmehr et al., 2010) identified a subset of 12 samples to have the glioblastoma CpG island methylator phenotype (G-CIMP). This independent assessment of G-CIMP subtype, using the clustering method applied by Noushmehr et al. in their original article, is used to assess our clustering results in the full feature set, as well as after non-specific filtering. (N=20,549 features total).

Data set #3 is HM450 data (plates 79, 111, and 130) of 99 glioblastoma cancers. Again, hierarchical cluster analysis of the CIMP-related features provided by Noushmehr et al. (Noushmehr et al., 2010) allowed us to identify a subset of G-CIMP samples in our data set (n=6). This independent assessment of G-CIMP subtype is used to assess our clustering results using the non-specific filtering methods described in this paper (N=374,601 features).

Data set #4 is HM27 data (plate 64) of 50 Kidney Renal Clear-cell (KIRC) cancer and 45 normal kidney tissues (N=21,624 features).

Data set #5 is HM450 data of 283 Kidney KIRC cancers and 160 normal kidney tissues (N=374,708 features).

Data set #6 is HM27 data (plate 93) of 37 infiltrating ductal carcinomas and 20 normal breast tissues (N=21,787 features).

Data set #7 is HM450 data (plate 109) of 56 infiltrating ductal carcinomas and 17 normal breast tissues (N=377,853 features).

Data set #8 is HM450 data of normal whole blood samples from NCBI’s Gene Expression Omnibus under accession number GSE40279 (Hannum et al., 2013). We restricted our analysis to the 84 samples on plate 2 with subjects’ ages ranging from 28 to 86 years (N=383,911 features).
