# Supplementary material for: Non-specific filtering of beta-distributed data
Source: BMC Bioinformatics. 2014 Jun 19;15:199. doi: 10.1186/1471-2105-15-199 (PMC4230495; doi:10.1186/1471-2105-15-199)
Supplement: Additional file 5: Table S1 — Area under the curve (95% confidence interval) for simulation results in Figure 2. [file 1471-2105-15-199-S5.docx]

# Additional file 5 – Supplemental Table 1

# Supplemental Table 1. Area under the curve (95% confidence interval) for simulation results in Figure 2.

|  | **Sample ratio of group1 : group2** | | |
| --- | --- | --- | --- |
| **Methods** | **180 non-CIMP:**  **20 CIMP** | **100 non-CIMP:**  **100 CIMP** | **20 non-CIMP:**  **180 CIMP** |
| SD-b | 0.52 (0.48, 0.57) | 0.54 (0.49, 0.58) | 0.51 (0.46, 0.56) |
| SD-m | 0.52 (0.48, 0.57) | 0.53 (0.48, 0.58) | 0.48 (0.44, 0.52) |
| MAD | 0.50 (0.46, 0.54) | 0.50 (0.46, 0.54) | 0.49 (0.45, 0.54) |
| DIP | 0.49 (0.45, 0.54) | 0.50 (0.45, 0.55) | 0.51 (0.47, 0.55) |
| Precision | 0.53 (0.48, 0.58) | 0.54 (0.49, 0.58) | 0.50 (0.45, 0.55) |
| **BQ-GOF** | **0.56 (0.52, 0.61)** | **0.63 (0.59, 0.68)** | **0.57 (0.53, 0.62)** |
| **TM-GOF** | **0.57 (0.53, 0.62)** | **0.70 (0.66, 0.75)** | **0.62 (0.57, 0.66)** |
| **TQ-GOF** | **0.59 (0.55, 0.63)** | **0.73 (0.68, 0.77)** | **0.63 (0.59, 0.68)** |
| **BR** | **0.58 (0.54, 0.62)** | **0.69 (0.64, 0.73)** | **0.63 (0.58, 0.68)** |
| **AR** | **0.58 (0.54, 0.62)** | **0.70 (0.65, 0.74)** | **0.64 (0.59, 0.68)** |
| **WAR** | **0.57 (0.53, 0.62)** | **0.66 (0.62, 0.71)** | **0.61 (0.56, 0.65)** |

# 
