# Supplementary material for: Non-specific filtering of beta-distributed data
Source: BMC Bioinformatics. 2014 Jun 19;15:199. doi: 10.1186/1471-2105-15-199 (PMC4230495; doi:10.1186/1471-2105-15-199)
Supplement: Additional file 6: Table S2 — Adjusted Rand Index of RPMM cluster analysis result using a variety of filtering methods for multiple data sets. [file 1471-2105-15-199-S6.docx]

# Additional file 6 – Supplemental Table 2

**Supplemental Table 2. Adjusted Rand Index of RPMM cluster analysis result using a variety of filtering methods for multiple data sets.**

|  | **Data set 1** | **Data set 2** | **Data set 3** | **Data set 4** | **Data set 5** | **Data set 6** | **Data set 7** |
| --- | --- | --- | --- | --- | --- | --- | --- |
| **Tissue type** | Colon cancer | Glioblastoma | Glioblastoma | Kidney | Kidney | Breast | Breast |
| **Platform** | HM27 | HM27 | HM450 | HM27 | HM450 | HM27 | HM450 |
| **# of probes after preprocessing** | 19965 | 20549 | 374601 | 21624 | 374708 | 21787 | 377853 |
| **# of samples** | 20 NONCIMP vs. 6 CIMP | 74 NONCIMP vs. 12 CIMP | 93 NONCIMP vs. 6 CIMP | 50 KIRC vs. 45 normal | 283 KIRC vs. 160 normal | 37 Breast cancer vs. 20 normal | 56 Breast cancer vs. 17 Normal |
| **No filter** | 0.12 | 0.22 | NA | 0.75 | NA | 0.71 | NA |
| **Filter top 1000 by:** |  |  |  |  |  |  |  |
| **Random *** | 0.07 | 0.21 | 0.003 | 0.74 | 0.46 | 0.64 | 0.27 |
| **SD-b** | 0.29 | 0.27 | 0.03 | 0.54 | 0.34 | 0.53 | 0.31 |
| **SD-m** | 0.29 | 0.27 | 0.08 | 0.67 | 0.31 | 0.49 | 0.35 |
| **MAD** | -0.10 | 0.21 | 0.02 | 0.55 | 0.32 | 0.48 | 0.31 |
| **DIP** | 0.45 | 0.00 | -0.04 | 0.54 | 0.33 | 0.52 | 0.31 |
| **Precision** | 0.32 | 0.34 | 0.09 | 0.52 | 0.25 | 0.52 | 0.29 |
| **BQ-GOF** | 0.27 | 0.22 | 0.15 | 0.88 | 0.31 | 0.42 | 0.27 |
| **TM-GOF** | 0.45 | 0.16 | 0.16 | 0.57 | 0.15 | 0.14 | 0.02 |
| **TQ-GOF** | 0.45 | 0.16 | 0.16 | 0.50 | 0.14 | 0.15 | 0.01 |
| **BR** | 0.29 | 0.20 | 0.13 | 0.65 | 0.30 | 0.47 | 0.27 |
| **AR** | 0.33 | 0.30 | 0.18 | 0.63 | 0.37 | 0.46 | 0.26 |
| **WAR** | 0.29 | 0.27 | 0.07 | 0.64 | 0.27 | 0.52 | 0.33 |
| **SD-b + TM-GOF**** | 0.30 | 0.29 | 0.08 | 0.67 | 0.38 | 0.46 | 0.21 |
| * Average adjusted rand index from 10 analyses of randomly sampled feature sets | | | |  |  |  |  |
| ** combine top 500 SD-b + top 500 TM-GOF features | | |  |  |  |  |  |
