# Supplementary material for: Non-specific filtering of beta-distributed data
Source: BMC Bioinformatics. 2014 Jun 19;15:199. doi: 10.1186/1471-2105-15-199 (PMC4230495; doi:10.1186/1471-2105-15-199)
Supplement: Additional file 7: Table S3 — Genomic context of the features selected by the top two filter methods. a. For HM27 platform (N/%). b. For HM450 platform (N/%). [file 1471-2105-15-199-S7.docx]

# Additional file 7 – Supplemental Table 3

**Supplemental Table 3a. For HM27 platform (N/%)**

|  |  |  | **Filter by SD-b** | | | | **Filter by TM-GOF** | | | |
| --- | --- | --- | --- | --- | --- | --- | --- | --- | --- | --- |
|  |  |  | **Data set 1** | **Data set 2** | **Data set 4** | **Data set 6** | **Data set 1** | **Data set 2** | **Data set 4** | **Data set 6** |
|  | **All CpG Targets** | **Filtered Set** | **Colon Cancer** | **Glioblastoma** | **Kidney Cancer vs Normal** | **Breast Cancer vs Normal** | **Colon Cancer** | **Glioblastoma** | **Kidney Cancer vs Normal** | **Breast Cancer vs Normal** |
| **total probes included** | 27578 | 22198 | 1000 | 1000 | 1000 | 1000 | 1000 | 1000 | 1000 | 1000 |
| **CpG island** | 41.8% | 46.5% | 75.4% | 59.0% | 21.9% | 33.8% | 74.4% | 73.8% | 79.6% | 66.9% |
| **promoter region** | 52.2% | 50.4% | 39.4% | 45.3% | 53.9% | 47.0% | 43.2% | 43.4% | 38.5% | 42.1% |
| **transcribed region** | 49.5% | 51.3% | 63.0% | 56.2% | 47.0% | 53.9% | 58.4% | 58.0% | 62.4% | 60.6% |
| **exonic region** | 25.4% | 26.9% | 37.5% | 31.5% | 27.5% | 31.3% | 33.5% | 31.8% | 37.8% | 34.2% |
| **intronic region** | 74.6% | 73.1% | 62.5% | 68.5% | 72.5% | 68.7% | 66.5% | 68.2% | 62.2% | 65.8% |

**Supplemental Table 3b. For HM450 platform (N/%)**

|  |  |  | **Filter by SD-b** | | | | **Filter by TM-GOF** | | | |
| --- | --- | --- | --- | --- | --- | --- | --- | --- | --- | --- |
|  |  |  | **Data set 3** | **Data set 5** | **Data set 7** | **Data set 8** | **Data set 3** | **Data set 5** | **Data set 7** | **Data set 8** |
|  | **All CpG Targets** | **Filtered Set** | **Glioblastoma** | **Kidney Cancer vs Normal** | **Breast Cancer vs Normal** | **Normal Blood** | **Glioblastoma** | **Kidney Cancer vs Normal** | **Breast Cancer vs Normal** | **Normal Blood** |
| **total probes included** | 482,421 | 384310 | 1000 | 1000 | 1000 | 1000 | 1000 | 1000 | 1000 | 1000 |
| **Type I design** | 28.1% | 29.2% | 56.3% | 22.2% | 36.0% | 31.0% | 49.4% | 27.8% | 41.8% | 12.8% |
| **Type II design** | 71.9% | 70.8% | 43.7% | 77.8% | 64.0% | 69.0% | 50.6% | 72.2% | 58.2% | 87.2% |
|  |  |  |  |  |  |  |  |  |  |  |
| **CGI Promoter** | 21.5% | 24.8% | 32.1% | 5.4% | 15.6% | 10.4% | 55.5% | 19.1% | 56.7% | 16.5% |
| **CGI Exon** | 2.5% | 3.0% | 5.4% | 2.0% | 2.8% | 4.3% | 3.0% | 4.6% | 2.9% | 2.8% |
| **CGI Intron** | 2.9% | 3.3% | 6.3% | 2.2% | 4.1% | 5.1% | 4.8% | 3.4% | 2.6% | 2.4% |
| **CGI Intergenic** | 4.2% | 4.8% | 14.1% | 2.7% | 9.0% | 12.1% | 9.2% | 4.5% | 4.8% | 5.1% |
| **non-CGI Promoter** | 22.2% | 21.6% | 16.2% | 20.7% | 20.2% | 19.4% | 9.3% | 15.3% | 14.6% | 20.8% |
| **non-CGI Exon** | 7.0% | 7.9% | 3.3% | 7.3% | 5.8% | 3.9% | 4.3% | 11.7% | 5.0% | 8.1% |
| **non-CGI Intron** | 20.9% | 18.9% | 8.3% | 33.1% | 21.4% | 20.4% | 9.9% | 23.7% | 9.5% | 27.0% |
| **non-CGI Intergenic** | 18.7% | 15.5% | 14.3% | 26.6% | 21.1% | 24.4% | 4.0% | 17.7% | 3.9% | 17.3% |
